# The role of oncostatin M receptor gene polymorphisms in bladder cancer

**DOI:** 10.1186/s12957-018-1555-7

**Published:** 2019-02-12

**Authors:** Shi Deng, Sheng yin He, Pan Zhao, Peng Zhang

**Affiliations:** Department of Urology, Institute of Urology, West China Hospital, Sichuan University, 37# Guoxuexiang Street, Chengdu, 610041 People’s Republic of China

**Keywords:** Oncostatin M receptor, Bladder cancer, Polymorphisms, Cancer risk

## Abstract

**Background:**

Oncostatin M receptor (*OSMR*) represents a part of the interleukin-six (IL6) cytokine group that was discovered recently to be closely associated with cell’s growth and differentiation, inflammation, and enhancement of metastatic capacity. A comprehensive study suggests a close relationship between *OSMR* and papillary thyroid cancer, colorectal cancer, breast cancer, and other tumors. However, the relationship between *OSMR* and bladder cancer has yet to be determined.

**Methods:**

Three hundred six patients (including 142 patients with muscle-invasive bladder cancer and 164 patients with non-muscle-invasive bladder cancer) as well as 459 normal controls were included in this study. Two tag SNPs of *OSMR*, rs2278329, and rs2292016 were genotyped by TaqMan® SNP Genotyping Assay method and then the associations with bladder cancer were analyzed, as well as risk factors and prognosis.

**Results:**

Patients with bladder cancer and controls did not differ significantly in terms of genotype frequencies and allele frequency distribution of rs2278329 (*P* = 0.77, OR = 0.97) and rs2292016 (*P* = 0.39, OR = 1.20) respectively. For rs2278329, no differences were found in terms of risk factors in stratified analyses. However, rs2292016 was associated with recurrence and tumor grade. GT/TT was found to increase the risk of relapse compared to the patients without allele T (GG genotype) (*P* = 0.016, OR = 1.878, 95% CI = 1.12–3.14) with the T allele of rs2292016 being a risk factor for recurrence (*P* = 0.032, OR = 0.67, 95% CI = 0.47–0.97). Besides, patients with GT genotype often present with high-grade bladder cancer (*P* = 0.003, OR = 2.33, 95% CI = 1.32–4.17). Multiple Cox regression analysis showed that rs2278329 and rs2292016 were related to the recurrence-free survival and overall survival in non muscle invasive bladder cancer (NMIBC) patients. For rs2278329, GA genotype could affect recurrence-free survival (*P* = 0.01, OR = 2.16, 95% CI = 1.17–3.98). For rs2292016, TT/GT genotype had a lower risk of death compared with GG homozygote genotype, and T was a protective factor for overall survival in bladder cancer (*P* = 0.029, OR = 0.22, 95% CI = 0.06–0.86).

**Conclusions:**

*OSMR* genotype frequencies were found to be associated with higher recurrence in bladder cancer, and it may serve as a biomarker candidate gene to predict prognosis of this disease. Further validation of OSMR as biomarker is required.

## Background

Cancer of the bladder is a frequently encountered malignancy. It is associated with a poor quality of life. According to the 2012 World Health Organization statistics, the number of new bladder cancer cases was approximately 429,000 worldwide, which ranks ninth in the incidence of malignant tumors. The number of deaths attributed to bladder cancer was approximately 165,000, ranking thirteenth among all malignant tumors [1]. In USA, an estimated 74,690 cases of bladder cancer occurred in 2014, including 65,390 males and 18,300 females [2]. In China, the incidence of bladder cancer was the tenth highest among malignant tumors, with increased death rate over the years 1991 to 2005 [3]. Bladder cancer comprises of muscle-invasive bladder cancer (MIBC) and non-muscle-invasive bladder cancer (NMIBC). Furthermore, during the initial diagnosis, most cases of bladder cancer belong to NMIBC, constituting nearly 70%, while MIBC accounts for approximately 25% [4]. After transurethral resection of bladder tumor (TURBT) combined with postoperative perfusion of chemotherapy, some patients will undergo tumor relapse, and nearly 10% of the NMIBC patients will progress to MIBC and even distant metastasis [5]. MIBC patients' experience low 5-year survival rates at 69%, with metastasis lowering this survival rate rapidly to 6% [4]. Early diagnosis and treatment of malignant tumors has a significant impact on the prognosis of patients. Tajuddin SM et al. found that if bladder cancer patients received the early diagnosis and treated at an early stage, the 5-year survival rate was nearly three times higher than the late stage [6].Until now, early diagnosis of bladder cancer has been accomplished using cystoscopy, imaging, and urinary cytology. Cystoscopy and pathological biopsy represent the gold standard for the diagnosis of bladder cancer. However, the disadvantages include high cost, trauma, pain, and the inability of these approaches to predict the treatment outcomes.

Bladder cancer has a complex etiology, which may be closely associated with chromosomal aberrations, gene mutations, lifestyles, and environmental factors. It is a pathological process mediated by multiple genes and affected by multiple factors. The study found that smoking or long-term exposure to chemical carcinogens are high risk factors leading to carcinogenesis of urothelial mucosa. However, only a small proportion of individuals exposed to these high risk factors eventually develop bladder cancer, highlighting the crucial contributions of genetic differences to the development of this disease [7]. Today, the role of genetic factors in the incidence of bladder cancer has attracted widespread attention and several genes are currently associated with the incidence of bladder cancer, including CD44, CRCC1, and PDCD6 [8–10]. However, none of these genes were recommended for bladder cancer detection and the study of other gene combination with existing genes might improve its clinical value to predict the occurrence and prognosis of bladder cancer.

Oncostatin M (*OSM*) is first discovered in1986 by Zarling et al. and is a member of interleukin-6 family [11, 12]. As a well-known multifunctional cytokine, *OSM* has multiple effects and functions, which are important in controlling immune responses, inflammation, cell proliferation, and tumor formation [13]. Current research indicates that there are two types of receptors for OSM, LIF binding protein and *OSM*-specific receptor (*OSMR*) [12]. *OSMR* receptor plays a key role in signal recognition. When *OSM* binds to *OSMR*, Janus protein tyrosine kinase (JAK) can be activated followed by activation of the mitogen-activated protein kinase (MAPK) as well as the signal transducer and activator of transcription (STAT, mainly STAT3) [14–16]. OSMR is active in a wide range of solid tumor cell lines, including breast tumor, colorectal cancer, bone carcinoma, and so on. West N et al. found that high OSMR was associated with reduced estrogen receptor alpha (ER), which could reduce the risk of tumor progression in breast tumor. Besides, patients with invasive breast cancer who had high OSMR expressions were previously observed to experience shorter overall survival and disease-free periods [17]. An investigation of OSMR gene methylation by Hibi K et al. in colorectal cancer patients revealed that OSMR methylation may prevent the tumorigenesis, progression in non-invasive colorectal cancer [18]. Stacey L et al. demonstrated that *OSMR* expression stimulated tumor angiogenesis and pro-invasive tumor activity in osteosarcoma cell lines, while David E et al. found *OSMR* is a growth factor for Ewing sarcoma [13, 19]. Recently, several gene polymorphisms (SNPs) were detected in the promoters regions of the *OSMR* gene and showed that the gene polymorphisms were associated with tumor, chronic inflammation [20, 21]. Unfortunately, studies have yet to delineate the relationship between *OSMR* SNPs and the initiation, progression, and prognosis of cancer of the bladder.

## Patients and methods

### Study subjects

Our investigation enrolled 306 cases of bladder cancer diagnosed between March 2009 to July 2014 in Sichuan University’s West China Hospital. All patients underwent surgery and pathologically confirmed as bladder cancer at the Department of Pathology in West China Hospital. None of the patients received neoadjuvant therapy including radiation and chemotherapy. The postoperative status of all the patients was successfully evaluated by phone, letter, or in an out-patient follow-up. The start date of the follow-up was recorded when the result was reported by the Department of Pathology, and the end date represented the last follow-up (July 2014), metastasis, or tumor-related death. The normal control group come from health examination center of our hospital represented the general population. A detailed analysis of the participants’ medical information ensured that the general population in the normal group had no history of malignant tumor, severe liver or kidney dysfunction, structural heart disease, diabetes, or diseases of the immune system. Finally, 459 individuals among the general population served as the normal control group. All the research subjects were Han residents of Sichuan province. Prior approval was obtained from the West China Hospital Ethics Committee while each participant gave signed informed consent.

### DNA extraction and genotyping

The current study involves genotyping of two single nucleotide polymorphisms (SNPs): rs2278329 and rs2292016 (-100G/T). With a DNA isolation kit supplied by Bioteke (Peking, China), EDTA-treated peripheral blood samples (200 μl) were used to extract individual genomic DNA. *OSMR* polymorphism genotyping was carried out with the TaqMan® SNP Genotyping Assay (Applied Biosystems, ABI, Foster City, CA) using Assay ID C__15965230_10 for rs2278329 and C__15966926_10 for rs2292016, respectively. SNPs were distinguished upon completion of real-time PCR using fluorescent VIC dye to label the allelic A probe for rs2278329 and allelic G probe for rs2292016 while others were labeled with fluorescent FAM dye. Real-time PCR using a TaqMan probe was performed with the following: a total of 5 μl mixture was used for each reaction and contained 2.5 μl 2× TaqMan Universal PCR Master Mix, 0.25 μl 20× SNP Genotyping Assay, and 10 ng of genomic DNA. Conditions for real-time PCR are as described: 95 °C for 10 min, followed by 45 cycles at 92 °C for 15 s, 60 °C for 1 min. Approximately 10% of the samples were selected randomly for repeated assays. All results showed 100% concordance.

### Statistical analyses

The SPSS version 13.0 (SPSS Inc., Chicago, IL, USA) was used to carry out statistical analyses. Direct counting was used to determine the frequencies of the two tag SNP genotypes. Hardy–Weinberg equilibrium was confirmed with a chi-square test. The differences between alleles and frequencies were analyzed using odds ratios (OR) and respective 95% confidence intervals (CI). SNP Stats was used to analyze genotypic variation in the genetic models. Statistical significance was interpreted when the *p* value was less than 0.05.

Univariate analysis of smoking status, gender, age, tumor stage and grade, along with genotypes and prognostic factors was conducted. Hazard ratios (HR) and 95% CIs were determined using Cox proportional hazards regression analysis.

## Results

### Genotype and allele frequencies of OSMR SNPs in bladder cancer patients and controls

Genotyping of the two SNPs were carried out in 459 controls and 306 patients that had bladder cancer. DNA sequencing analyzing was used to confirm the identities of three genotypes of each SNP. All the observed genotype frequencies in patients’ bladder cancer and controls were consistent with the predicted values under Hardy–Weinberg equilibrium. The age, gender, smoking status, and other pathological features (clinical stage, tumor grade) of the two groups were compared; no statistically significant difference between groups was  detected (Table 1). Table 2 depicts the allele frequencies and distribution of genotypes of *OSMR* SNPs rs2278329 and rs2292016 in patients with bladder cancer and controls. These factors did not differ significantly between the two groups.

**Table 1:** Characteristics of the study population

	NMIBC group	MIBC group	Controls
	*N* = 164 (%)	*N* = 142 (%)	*N* = 459 (%)
Sex			
Male	128 (78.0)	113 (79.6)	355 (76.4)
Female	36 (22.0)	29 (20.4)	104 (23.6)
Age at first diagnosis (mean ± SD)	61.8 ± 12.7	65.9 ± 10.8	64.6 ± 5.7
Smoking status			
Smokers	84 (51.2)	75 (52.8)	214 (46.6)
Non-smokers	80 (48.8)	67 (47.2)	245 (53.4)
Clinical stage			
Ta	10 (6.1)	–	–
T1	154 (93.9)	–	–
T2	–	87 (61.3)	–
T3a	–	31 (21.8)	–
T3b	–	13 (9.2)	–
T4	–	11 (7.7)	–
Tumor grade			
Low grade	109 (66.5)	23 (16.2)	–
High grade	55 (33.5)	119 (83.8)	–

**Table 2:** Distribution of SNPs in *OSMR* among patients and controls and their association with bladder cancer risk

Model		rs2278329					rs2292016			
	Genotype	Controls	Patients	OR (95% CI)	*P* value	Genotype	Controls	Patients	OR (95% CI)	*P* value
		*N* = 459 (%)	*N* = 306 (%)				*N* = 459 (%)	*N* = 306 (%)		
Codominant	GG	207 (45.1)	133 (43.5)	1.00 (reference)	0.88	GG	191 (41.6)	136 (44.4%)	1.00 (reference)	0.70
	GA	200 (43.6)	139 (45.4)	0.92 (0.68–1.26)		GT	209 (45.5)	135 (44.1)	1.10 (0.81–1.50)	
	AA	52 (11.3)	34 (11.1%)	1.23 (0.79–1.89)		TT	59 (12.8)	35 (11.4)	1.20 (0.75–1.93)	
Dominant	GG	207 (45.1)	133 (43.5)	1.00 (reference)	0.66	GG	191 (41.6)	136 (44.4)	1.00 (reference)	0.44
	GA/AA	252 (54.9)	173 (56.5)	0.94 (0.70–1.25)		GT/TT	268 (58.4)	170 (55.6)	1.12 (0.84–1.50)	
Recessive	GG/GA	407 (88.7)	272 (88.9%)	1.00 (reference)	0.93	GG/GT	400 (87.2)	271 (88.6)	1.00 (reference)	0.56
	AA	52 (11.3)	34 (11.1%)	1.02 (0.65–1.62)		TT	59 (12.8)	35 (11.4)	1.14 (0.73–1.78)	
Overdominant	GG/AA	259 (56.4)	167 (54.6%)	1.00 (reference)	0.61	GG/TT	250 (54.5)	171 (55.9)	1.00 (reference)	0.70
	GA	139 (45.4)	139 (45.4%)	0.93 (0.69–1.24)		GT	209 (45.5)	135 (44.1)	1.06 (0.79–1.42)	
	Allele									
	G	614 (66.9)	405 (66.2)	0.97 (0.78–1.20)	0.77	G	591 (64.4)	407 (66.5)	1.20 (0.84–1.70)	0.39
	A	304 (33.1)	207 (33.8)			T	327 (35.6)	205 (33.5)		

*N* corresponds to the number of individuals

### OSMR tag SNPs and patients’ characteristics

*OSMR* SNPs effects on the development of bladder malignancies were further explored using stratified analyses. Genotype distributions of *OSMR* tag SNPs in different groups stratified according to patient demographics including smoking, gender, age, tumor grade and stage, as well as recurrence of disease are displayed in Tables 3 and 4. For rs2278329, the results showed it is irrelevant to smoking, gender, age, tumor grade and stage, as well as the recurrence status in bladder cancer. For rs2292016, significant differences in allele and genotype frequency distributions were observed in patients with bladder malignancy. In the dominant model, patients with GT/TT had a higher risk of relapse in contrast to patients without allele T (GG genotype) (*P* = 0.016, OR = 1.878, 95% CI = 1.12–3.14). The allele T of rs2292016 is a risk factor for bladder cancer recurrence (*P* = 0.032, OR = 0.67, 95% CI = 0.47–0.97). Additionally, GT heterozygote of rs2292016 genotype had higher risk of developing high-grade bladder cancer in contrast to the homozygote genotype (GG/TT) in the overdominant model (*P* = 0.003, OR = 2.33, 95% CI = 1.32–4.17).

**Table 3:** Association between clinical characteristics of patients with bladder cancer and rs2278329

Characteristics	Cases No. (%)	Genotype No. (%)			*P* value	Allele No. (%)		*P* value	OR (95%CI)
		GG	GA	AA		G	A		
Gender									
Male	241 (78.8)	104 (43.1)	110 (45.6)	27 (11.2)	0.62	318 (66.0)	164 (34.0)	0.84	1.04 (0.69–1.57)
Female	65 (21.2)	29 (44.6)	29 (44.6)	7 (10.8)		87 (66.9)	43 (33.1)		
Age									
≤ 64	139 (45.4)	61 (43.9)	61 (43.9)	17 (12.2)	0.92	183 (65.8)	95 (34.2)	0.87	1.03 (0.74–1.44)
> 64	167 (54.6)	72 (43.1)	78 (46.7)	17 (10.2)		222 (66.5)	112 (33.5)		
Smoking status									
Non-smokers	147 (48.0)	58 (39.5)	69 (46.9)	20 (13.6)	0.16	185 (62.9)	109 (37.1)	0.1	1.32 (0.95–1.85)
Smokers	159 (52.0)	75 (47.2)	70 (44.0)	14 (8.8)		220 (69.2)	98 (30.8)		
Stage									
NMIBC	164 (53.6)	71 (43.3)	73 (44.5)	20 (12.2)	0.80	215 (65.5)	113 (34.5)	0.97	1.00 (0.72–1.41)
MIBC	142 (46.4)	62 (43.7)	66 (46.5)	14 (9.9)		190 (66.9)	94 (33.1)		
Grade									
Low	132 (43.1)	57 (43.2)	56 (42.4)	19 (14.4)	0.4	170 (64.3)	94 (34.8)	0.42	1.15 (0.82–1.61)
High	174 (56.9)	76 (43.7)	83 (47.7)	15 (8.6)		235 (67.5)	113 (32.5)		
Recurrence									
Negative	217 (70.9)	99 (45.9)	94 (43.3)	24 (11.1)	0.54	292 (67.3)	142 (32.7)	0.37	0.85 (0.59–1.22)
Positive	89 (29.1)	34 (38.2)	45 (50.6)	10 (11.2)		113 (63.5)	65 (36.5)

**Table 4:** Association between clinical characteristics of patients with bladder cancer and rs2292016

Characteristics	Cases No.	Genotype No. (%)			*P* value	Allele No. (%)		*P* value	OR (95%CI)
		GG	GT	TT		G	T		
Gender									
Male	241 (78.8)	104 (43.1)	108 (44.8)	29 (12.0)	0.43	316 (65.6)	166 (34.4)	0.34	1.23 (0.81–1.87)
Female	65 (21.2)	32 (49.2)	27 (41.5)	6 (9.2)		91 (70.0)	39 (30.0)		
Age									
≤ 64	139 (45.4)	61 (43.9)	63 (45.3)	15 (10.8)	0.65	185 (66.5)	93 (33.5)	0.98	0.99 (0.71–1.40)
> 64	167 (54.6)	75 (44.9)	72 (43.1)	20 (12.0)		222 (66.5)	112 (33.5)		
Smoking status									
Non-smokers	147 (48.0)	65 (44.2)	62 (42.2)	20 (13.6)	0.35	192 (65.3)	102 (34.7)	0.55	1.11 (0.79–1.55)
Smokers	159 (52.0)	71 (44.6)	73 (45.9)	15 (9.4)		215 (67.6)	103 (32.4)		
Stage									
NMIBC	164 (53.6)	75 (45.7)	69 (42.1)	20 (12.2)	0.72	219 (66.8)	109 (33.2)	0.88	0.98 (0.70–1.37)
MIBC	142 (46.4)	61 (43.0)	66 (46.5)	15 (10.6)		188 (66.2)	96 (33.8)		
Grade									
Low	132 (43.1)	65 (49.2)	47 (35.6)	20 (15.2)	*0*.*009*	177 (67.0)	87 (33.0)	0.81	0.96 (0.68–1.35)
High	174 (56.9)	71 (40.8)	88 (50.6)	15 (8.6)		230 (66.1)	118 (33.9)		
Recurrence									
Negative	217 (70.9)	106 (48.9)	88 (40.5)	23 (10.6)	*0*.*036*	300 (69.1)	134 (30.9)	*0*.*032*	*0*.*67* (*0*.*47*–*0*.*97*)
Positive	89 (29.1)	30 (33.7)	47 (52.8)	12 (13.5)		107 (60.1)	71 (39.9)		

Values in italics are statistically significant

### OSMR tag SNPs and patients’ outcome

A total of 306 patients with bladder cancer were included in this investigation. After an average follow-up of 5 years, a total of 48 patients (NMIBC: 12/164, MIBC: 36/142) died, 89 patients (NMIBC: 45/164, MIBC: 44/142) again experienced the relapse, and 258 were patients alive (NMIBC: 152/164, MIBC: 106/142). Cox regression analysis adjusted by age, gender, smoking history, grading, and metastasis showed *OSMR* genotypes (rs2278329 and rs2292016) were related to the overall survival as well as the recurrence-free survival in NMIBC patients, not in MIBC patients (Table 5).

**Table 5:** Association between SNPs in *OSMR* and patient’s survival

SNP/genotype	NMIBC					MIBC						
	Alive/dead	HR (95% CI) ^a^	*P*	Recurrence/Non-recurrence	HR (95% CI) ^b^	*P*	Alive/dead	HR (95% CI) ^a^	*P*	Recurrence/Non-recurrence	HR (95% CI) ^b^	*P*
rs2278329												
GG	63/8			16/55			46/16			6/8		
GA	70/3			25/48			49/17			20/46		
AA	19/1			4/16			11/3			18/44		
Dominant		0.31 (0.09–1.06)	0.06		1.56 (0.0.84–2.89)	0.16		1.14 (0.57–2.26)	0.71		1.05 (0.56–1.96)	0.88
Recessive		0.77 (0.10–6.10)	0.80		0.49 (0.17–1.39)	0.18		1.10 (0.31–3.94)	0.88		1.54 (0.60–3.97)	0.37
Overdominant		0.31 (0.08–1.20)	0.09		*2*.*16* (*1*.*17*–*3*.*98*)	*0*.*01*		1.10 (0.57–2.13)	0.78		0.89 (0.49–1.63)	0.71
rs2292016												
GG	66/9			15/60			48/13			15/46		
GT	66/3			23/46			47/19			24/42		
TT	20/0			7/13			11/4			5/10		
Dominant		*0*.*22* (*0*.*06*–0.86)	*0*.*029*		1.74 (0.92–3.27)	0.08		1.36 (0.69–2.70)	0.38		1.45 (0.78–2.72)	0.24
Recessive		NA (0–NA)	0.98		1.18 (0.51–2.71)	0.70		1.21 (0.43–3.52)	0.72		1.12 (0.36–2.88)	0.82
Overdominant		0.37 (0.09–1.43)	0.15		1.58 (0.86–2.90)	0.14		1.25 (0.65–2.43)	0.50		1.37 (0.75–2.52)	0.30

*N* corresponds to the number of individuals

^a^Adjusted by age, sex, smoking status, tumor stage, and recurrence

^b^Adjusted by age, sex, smoking status, and tumor stage. Italicized values indicate a significant difference at the 5% level

**Fig. 1:**
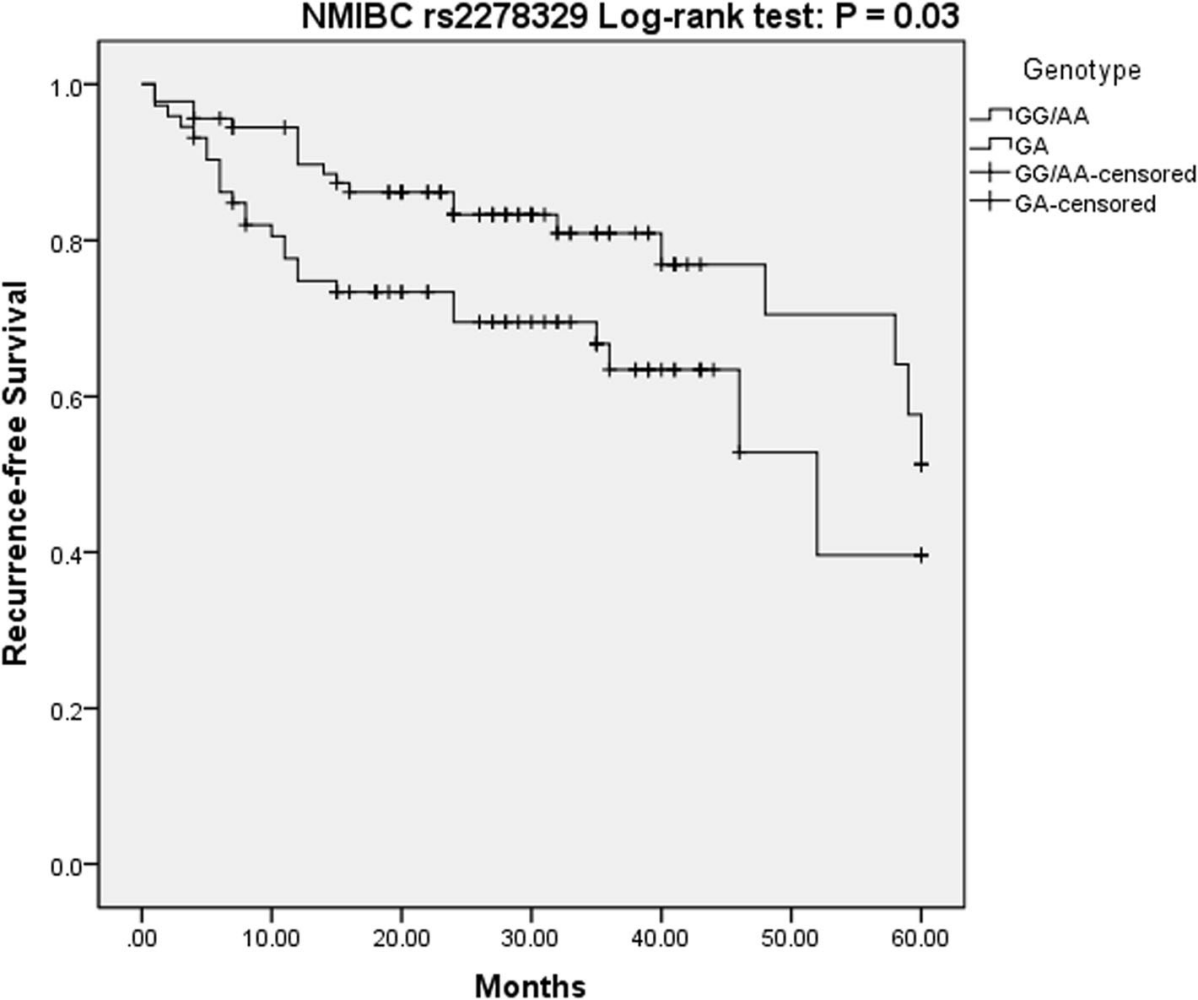
Kaplan Meier Survival curves for NMIBC patients on rs2278329

**Fig. 2:**
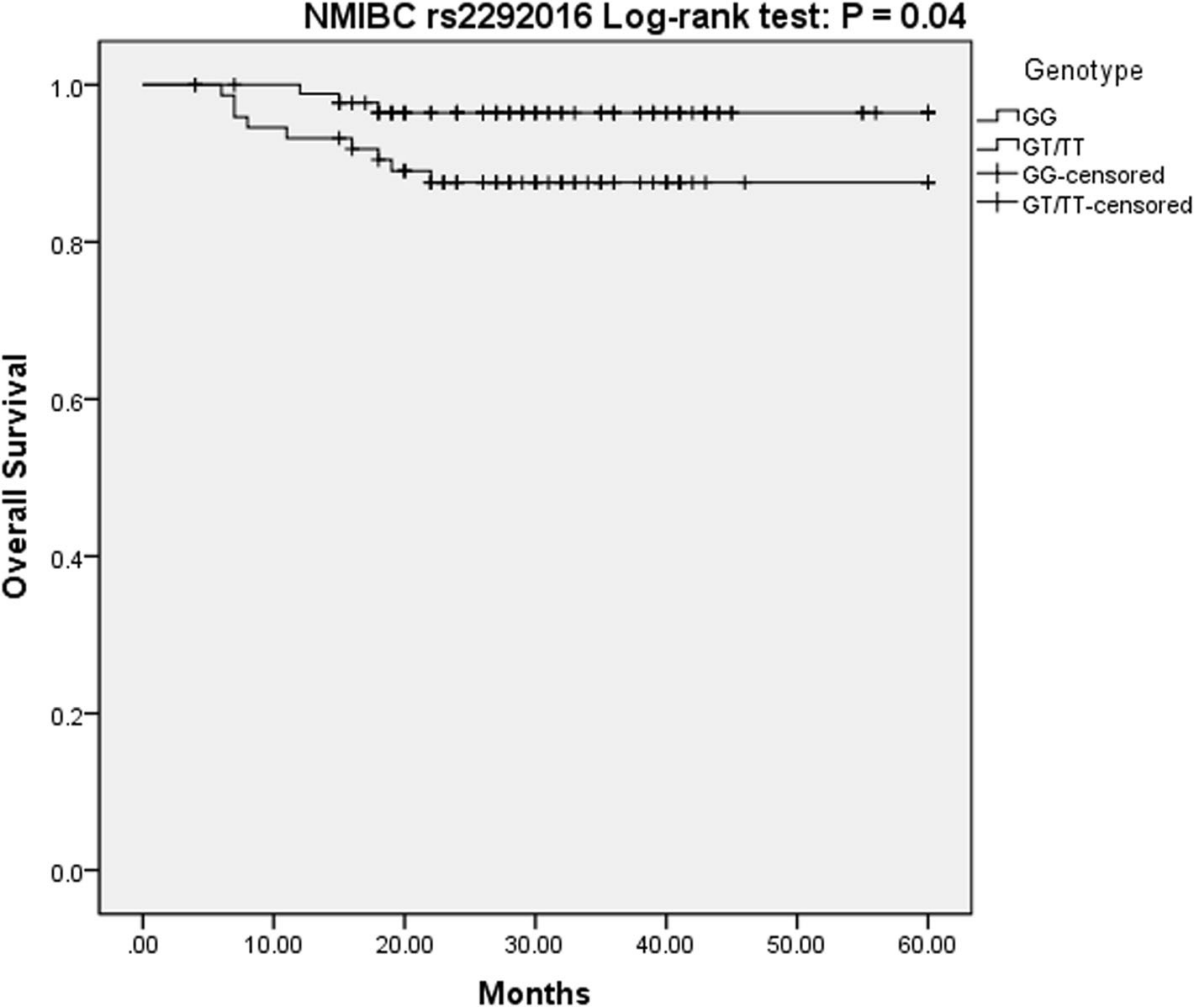
Kaplan Meier Survival curves for NMIBC patients on rs2292016

What is noteworthy, in patients with NMIBC, having the rs2278329 genotype may be an independent contributing determinant for recurrence-free survival. Based on the overdominance model, the GA genotype would affect the recurrence risk (*P* = 0.01, OR = 2.16, 95% CI = 1.17–3.98) (Fig. 1). However, overall survival in NMIBC patients, not the recurrence-free survival, was associated with rs2292016 genotype. Patients with (TT/GT) genotype had a lower risk of death compared with GG homozygote genotype in the dominant model, allowing us to conclude that the T allele may be an protective factor in NMIBC patients (*P* = 0.029, OR = 0.22, 95% CI = 0.06–0.86) (Fig. 2).

## Discussion

Gene polymorphism may be an adaptive manifestation in humans to the various risk factors during evolution, suggesting that similar diseases may have different clinical manifestations and similar treatment may possibly with varying efficacy in individuals. A serial of studies demonstrate that the onset and progression of bladder cancer are closely associated with multiple gene polymorphisms including CD44, XRCC1, and PDCD6 [8–10]. In this study, no association were found between bladder cancer patients and controls with respect to the genotypic and allelic frequencies of *OSMR* gene polymorphisms (rs2278329 and rs2292016). However, further analysis showed the rs2292016 was associated with clinicopathologic characteristics, such as higher tumor grade and recurrence. In addition, multivariate analysis indicated that recurrence-free survival was strongly linked to the rs2278329 genotype while overall survival depended on the presence of a rs2292016 genotype. Our study is the first of its kind to report a relationship between the *OSMR* gene and bladder cancer.

According to the tumor number, size, recurrence status, concomitant CIS or not, and tumor grade, NMIBC was divided into three risk groups based on the European Organization for Research and Treatment of Cancer (EORTC) [22]. Patient-derived data indicates that patients in the high-risk group may face high incidence of tumor recurrence and progression possibly because of the different pathophysiologic mechanism. Bladder tumor has a high possibility of postoperative recurrence, necessitating careful long-term clinical follow-up. Although cystoscopy, ultrasonic examination, urine cytology, etc. are useful tools to assess and monitor the relapses of bladder tumor, not all examination techniques are perfect. For example, cystoscopy is invasive thus unacceptable to many patients [23] while urine cytology is less sensitive especially for the low-grade bladder tumors [24]. With the gradual improvement of the diagnostic value of biological tests, serology, and molecular biology, serum tumor markers could be a new way to monitor tumor relapse. Nuclear Matrix Protein 22 (NMP22), bladder tumor antigen (BTA), and HA are among a few examples of bladder tumor markers. However, there are still many shortcomings of tumor markers, requiring the combination of other methods in order to provide an adequately sensitive and specific means for early diagnosis and postoperative surveillance [25, 26]. The results showed a close relationship between SNP rs2292016 and relapse. In this study, we found that rs2292016 T allele and GT/TT genotype in the dominant model were risk factors for recurrence and careful follow-up was needed in these patients.

Although the prognosis is generally good for most NMIBC patients, about 30–80% of patients may suffer a relapse and approximately 1–45% patients progress to MIBC in 5 years [22, 27, 28]. The treatment options of the NMIBC patients with high tumor grade have long been a contentious issue, especially the T1G3. For instance, Denzinger et al. found that T1G3 patients who received early cystectomy had a better 10-year CSS (78%) compared to those who received deferred cystectomy (51%) [29]. Similar conclusions were concluded from Herr et al. and Hautmann RE et al. [30, 31]. There are several potential explanations for this phenomenon. The repeated use of TUR in the NMIBC may improve the tumor staging and deferred cystectomy has been associated with increase in the prevalence of lymph node metastases (LNMs) [32]. In a retrospective study of 866 bladder tumor patients, Wiesner C et al. found that 8% of patients who had undergone a single TUR had LNMs, and the statistic increased threefold to 24% in those who had undergone 2–4 TURs [33]. In the overdominant model of this study, high-grade bladder cancers were more likely to be GT heterozygotes of the rs2292016 genotype rather than the homozygote genotype (GG/TT), indicating that patients with this specific genotype should consider undergoing early cystectomy.

*OSM*–*OSMR* signaling is a significant mediator of inflammatory disease, cell development, and is also considered an important mechanism in tumor progression [16]. Studies showed that *OSMR* is expressed by multiple neoplastic cells and tumor tissues, but the exact *OSM*–*OSMR* mechanisms seem to be influenced by cell-type and tumor types. In AIDS-related Kaposi’s sarcoma, *OSM* promoted the tumor proliferation and protein synthesis [34]. A similar phenomenon was observed in myeloma and human prostate cancer cells [35, 36]. In contrast, Hibi K et al. found *OSMR* to be more methylated in non-invasive colorectal cancer compared to invasive colorectal cancer. The highlights the tumor-suppressive role of *OSMR* in colorectal carcinoma [18]. Survivin, Mcl-1, and VEGF are examples of tumor-related factors whose expressions are thought to be regulated by STAT3. As stated before, *OSMR* was associated with activation of STAT3 signal transduction pathway. These all reveal that *OSMR* plays a role in tumor metabolism and possibly affects the chances of survival. Our study showed that rs2278329 and rs2292016 were associated with recurrence-free survival and overall survival in NMIBC patients. rs2278329 GA heterozygotes might have shorter durations of recurrence-free survival, while T alleles of rs2292016 was associated with increased survival. Patients with (TT/GT) genotype had a lower risk of death compared to those with the GG homozygote genotype*.* Based on the evidence above, we can draw a conclusion that OSMR decreases survival rate for bladder cancer.

## Conclusion

Our study shows a significant association between *OSMR* gene polymorphism and bladder cancer that potentially impacts tumor grade, recurrence, and overall survival. This is a first report of the association between bladder cancer and *OSMR* gene polymorphisms, to the best of our knowledge. However, this study still has its limitations. First, multiple genes have emerged as potentially having links with the occurrence, progression, and prognosis of bladder cancer. However, this study only evaluated the *OSMR* gene and there could be other genes that could synergistically affect the survival. Secondly, the tumors caused by genetic polymorphisms occur across different races, whereas the study participants represented only the Han residents of Sichuan province. Therefore, the study findings cannot be generalized or applicable to all the races. Furthermore, the study sample size was small, suggesting the need for larger studies to corroborate the results.

## Abbreviations

MIBC: Muscle-invasive bladder cancer
NMIBC: Non-muscle-invasive bladder cancer
STAT3: Signal transducer and activator of transcription 3
TURBT: Transurethral resection of bladder tumor
